# Development of a Novel Drug Delivery System “Nanoemulfoam” for Topical Delivery of Terbinafine Hydrochloride as a Repurposed Therapy in Skin Cancer: Formulation, Optimization, In Vitro Characterization, Ex Vivo Transdermal Permeability, Cytotoxicity Studies, and In Silico Assessment

**DOI:** 10.3390/ph18070972

**Published:** 2025-06-27

**Authors:** Abeer A. Musallam, Reem A. Aldeeb, Riham M. Mansour, Manar Abd El-karim Kassem, Doaa Fayez Saeed, Mahmoud A. Mahdy, Rana M. Abdelnaby, Hanan M. Elnahas, Tarek M. Ibrahim

**Affiliations:** 1 Department of Pharmaceutics, College of Pharmaceutical Sciences and Drug Manufacturing, Misr University for Science and Technology, 6th of October City, Giza 12582, Egypt; 2 Department of Pharmacology and Toxicology, College of Pharmaceutical Sciences and Drug Manufacturing, Misr University for Science and Technology, 6th of October City, Giza 12582, Egypt; 3 Department of Pharmaceutical Chemistry, College of Pharmaceutical Sciences and Drug Manufacturing, Misr University for Science and Technology, 6th of October City, Giza 12582, Egypt; 4 Department of Biochemistry, College of Pharmaceutical Sciences and Drug Manufacturing, Misr University for Science and Technology, 6th of October City, Giza 12582, Egypt; 5 Department of Pharmaceutics, Faculty of Pharmacy, Zagazig University, Zagazig 44519, Egypthananelnahas@gmail.com (H.M.E.);; 6 Department of Pharmaceutical Chemistry, Faculty of Pharmacy, Heliopolis University, Cairo 11785, Egypt

**Keywords:** terbinafine hydrochloride, nanoemulfoam, D-optimal design, drug repurposing, ex vivo permeation, cell viability, skin cancer

## Abstract

**Background:** Skin cancer has become a global health issue because of increasing exposure to environmental contaminants and UV radiation. Terbinafine hydrochloride (TRB), a broad-spectrum antifungal medication, has demonstrated notable anti-tumor properties in previous studies; however, its repurposing for skin cancer therapy remains underexplored. **Objective:** This study reports for the first time, the development of a new delivery system: a nanoemulsion (NE)–foam hybrid system, i.e., “nanoemulfoam” (NEF), designed to enhance the topical TRB delivery to the skin. The study applied this new hybrid system on TRB for managing skin cancer. **Method:** The TRB-loaded NEF was produced by loading TRB into a liquid NE. then this was incorporated into a liquid foam base and actuated into foam using a non-propellant mechanism. The NE was developed utilizing peppermint oil as the oil phase and Tween-20/ethanol as the surfactant/co-surfactant combination (Smix). The formulation underwent optimization using the D-optimal design that enabled the simultaneous evaluation of the impact of oil concentration and Tween 20 concentration in the Smix on the particle size (PS), zeta potential (ZP), and dissolution efficiency percent (DE%). **Results:** The optimal NE formula displayed a small PS of 186.60 ± 2.84 nm, ZP of −13.90 ± 0.99 mV, and DE% of 68.50 ± 1.78% (mean ± SD, *n* = 3). After incorporation into the foam system, the produced TRB-loaded NEF demonstrated a 7.43-fold increase in the drug transdermal flux in comparison with plain drug foam (*p* < 0.05). The TRB-loaded NEF showed no signs of inflammation or irritation when applied to abdominal rabbit skin, indicating its safety. The optimum formula exhibited a statistically significant 10-fold increase in cytotoxicity against A-431 skin cancer cells compared to TRB alone, along with a 1.54-fold increase in apoptosis (*p* < 0.05). Molecular docking studies targeting CDK2, a key regulator of cell proliferation and a known TRB target, revealed that TRB displayed highly favorable binding scores compared to the reference drug. **Conclusions:** The TRB-loaded NEF represents a promising nanotechnology-based approach for the topical treatment of skin cancer, supporting further investigation toward clinical translation.

## 1. Introduction

Skin cancer is the most prevalent kind of cancer globally among patients with fair skin. The skin cancer population is comprised of melanoma and non-melanoma skin cancers, while non-melanoma includes basal and squamous cell carcinoma [1]. The majority of cancer-related fatalities are attributed to melanoma, while non-melanoma is often accompanied by a benign progression and locally aggressive attributes, having the potential to cause substantial deformity [2]. In 2022, there were approximately 6.6 million new cases of skin cancer worldwide, with non-melanoma skin cancers accounting for about 6.3 million of these cases. Melanoma, while less common, is notably more lethal; an estimated 331,722 new melanoma cases were diagnosed globally in 2022, resulting in approximately 58,667 deaths [3]. This may result in detrimental physical and psychological effects on the individuals afflicted [4]. The prevalence of skin cancer is quickly growing globally, underscoring the need for more effective and safe therapy [5]. The establishment of such therapy still faces significant obstacles. Although chemotherapy, radiation therapy, and surgery are often commonly used treatments, they are linked to serious side effects and limited efficacy in advanced instances [6]. Accordingly, there is an increasing need for innovative treatment approaches that may enhance patient outcomes.

Scientists are now investigating a unique technique that may directly target tumors. One very promising approach includes integrating drugs into nanocarriers [5]. This approach has multiple benefits, including increased therapeutic effectiveness, decreased toxicity, enhanced drug solubility, and targeted drug delivery to specific tumor cells [7,8]. Among different varieties of nanoformulations is the nanoemulsion (NE), which comprises dispersions of nanoscale droplets of one liquid colloidally suspended into another [5]. NE can encapsulate medications that have a lipophilic nature, hence allowing the production of aqueous formulations that promote targeted cell delivery [9]. The targeting of drugs to cancer cells can increase the efficiency of cancer therapy and limit harm to normal cells. Additionally, NE may help enhance the permeation and retention of medications in the skin, and this will be very beneficial in skin cancer treatment [10].

Another approach is to repurpose/reposition existing medications for new uses. This technique entails discovering new applications for drugs that have already been authorized for other indications [11,12]. The process of novel medication development within the pharmaceutical sector is complicated and protracted. Before medicine is marketed, the Food and Drug Administration (FDA) lays out a four-stage process involving preclinical research, development, clinical trials, and discovery. According to the FDA, each of the phases lasts between 12 and 15 years [13]. Drug repositioning has been shown to be a useful method in pharmaceutical discovery due to cost reduction, accelerated development cycles, and increased probability of succeeding clinical trials, depending on the pre-existing pharmacokinetic attributes of the repositioned drug [5]. In this light, antifungal medication repositioning is attracting interest as a possible cancer therapy [14].

Terbinafine hydrochloride (TRB) is an antifungal drug that can be administered by topical or oral routes [6]. It functions by inhibiting fungal squalene oxidase, which promotes the synthesis of ergosterols inside fungi. This restriction permits squalene oxidase accumulation inside the fungi, resulting in their destruction [15]. TRB has shown a favorable safety profile with minimal incidence of pharmaceutical interactions. According to the study performed by C. S. Huang et al. (2008), TRB showed some potential anti-cancer activity for skin cancer treatment [14]. The TRB’s cytotoxic effect on cancer cells was attributed to its ability to block the squalene epoxidase enzyme. It was previously discovered that squalene epoxidase can reduce cell apoptosis and accelerate the cell cycle advancement in cancer cells, while inhibition of this enzyme might halt the cancer cells’ progression [16].

Foams are described as two-phase colloidal systems that resemble closed gas bubble agglomerations separated by a thin layer, with the absence of any connecting channels among them [17]. Foams have lately been identified as the ideal medium for skin problems in comparison to other traditional formulations. Due to their light consistency and high spreadability, they provide simplicity of application in an effortless manner by spreading with rubbing minimization, which is preferred in contact with irritated or sensitive skin [12]. Liquid foams are regarded as feasible and widely recognized drug delivery systems that play a significant role in topical medication administration [17]. Farkas et al. (2019) described two methodologies used in the foam system’s generation: gas supersaturation, which produces aerosol foam, and straightforward mechanical shaking, which induces foaming following actuation via a non-propellant pump mechanism [18].

Generally, the inclusion of ethanol in the topical formulation may result in dryness and loss of water of the skin owing to the alcohol’s defatting impact. This has cleared the way for the establishment of a new type of foam, known as oil/emulsion foams, which we named nanoemulfoam (NEF). These foams minimize water loss by refatting the skin. They boost the hydration in the stratum corneum, which strengthens the skin barrier function [19]. Foam formulations produced from NE (droplet size ranging from 20 to 200 nm) have great potential due to their capacity to solubilize difficult-to-dissolve active compounds, hence augmenting their bioavailability and effectiveness [18].

While TRB’s anticancer potential has been noted, it has limitations in drug delivery, particularly in achieving sufficient skin penetration and localizing therapeutic concentrations at the tumor site. To the authors’ knowledge, the NEF hybrid system was not published before. Therefore, the goal of this work was to integrate the NE into a foam system to develop a new NEF formulation for repositioning TRB to enhance dermal delivery, improve cytotoxic activity, and support the repurposing of TRB as a topical therapy to suppress carcinogenesis in human epithelioid squamous carcinoma (A-431) cells. The D-optimal design was utilized to find the optimum environment for formulating TRB-loaded NE and to develop the best formula with the favorable goals. The formula showing a small particle size (PS) with maximized zeta potential (ZP) and dissolution efficiency percent (DE%) was selected. The optimal formula was then characterized by its physicochemical properties. Following integration into a foam system, it was subjected to physicochemical, skin permeation, and histopathology tests. Furthermore, the optimal formula’s cell cytotoxicity, cell apoptosis, and cell cycle studies were evaluated to determine its potential to trigger programmable cell death. Finally, molecular docking investigation and target prediction analyses were carried out to further comprehend the TRB cytotoxic mechanism in the A-431 cell line.

## 2. Results and Discussion

### 2.1. Solubility Study of TRB in NE Formulation Constituents

**Figure 1:**
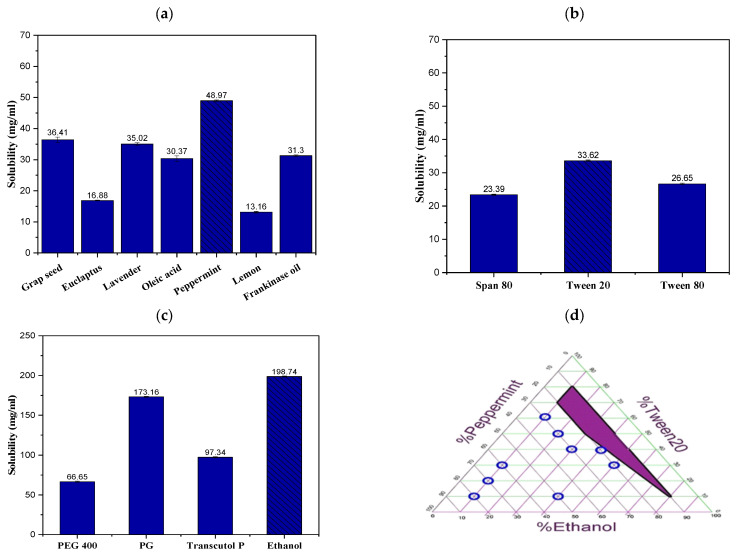
Solubility study of TRB in various (**a**) oils, (**b**) surfactants, and (**c**) co-solvents. (**d**) Ternary phase diagram of NE systems. The purple area refers to clear NE formulations, while the blue circles represent bluish-white microemulsion formulations.

Choosing the appropriate oily phase is crucial as it impacts the drug payload and its ability to remain soluble in the NE system [20]. Thus, the TRB’s solubility was tested in a variety of oils. As shown in Figure 1a, the greatest solubility of TRB was detected in peppermint oil (48.97 ± 0.22 mg/mL), while the reported minimum TRB solubility was in lemon oil (13.16 ± 0.012 mg/mL). As a result, peppermint oil was selected for further studies.

The selection of the surfactants and co-surfactants is also crucial, as they reduce the interfacial tension in the NE by being adsorbed at the oil/water interface and serving as an obstacle to hinder coalescence [21]. The choice of non-ionic surfactants is often favored over ionic surfactants because the latter frequently have bioactivity and toxicity concerns. Moreover, the hydrophilic nature of non-ionic surfactants renders them safer [22]. Thus, the solubilization capacity of non-ionic surfactants was investigated. Figure 1b shows that the tested surfactants had the highest solubilization power with Tween 20 (33.62 ± 0.23 mg/mL), followed by Tween 80 and Span 80, respectively. Accordingly, Tween 20 was selected for further investigation.

Co-surfactants are used in NE systems to reduce the surfactant concentrations. The co-surfactant is often employed to minimize the stress during bending by turning the interfacial membrane malleable, allowing it to deform to effectively cover each globule easily [20]. Various co-surfactants were evaluated for their solubilization capacity in this research. Out of the assessed co-surfactants, ethanol was chosen for the formulation of NE due to its highest drug solubilization power of 198.74 ± 0.68 mg/mL (Figure 1c).

### 2.2. Construction of the Ternary Phase Diagram

The phase diagram illustrates the correlation between the composition of NE and its physical appearance. It was noticed that the Smix ratio significantly affected the solubility of the oily phase and decreased the system’s free energy [21]. The graph was generated using peppermint oil, Tween 20, and ethanol at various ratios without drug addition to determine the exact concentration ratios of the components. Based on thorough visual inspection, the shaded portion in Figure 1d indicates the clear and readily flowable NE regions subsequent to dilution of various preparations. In contrast, the non-shaded parts depict the milky/multiphase macroemulsion regions. A bluish-white appearance can also be observed at specific points that indicate the formation of microemulsion systems. The preparations that show a clear appearance were chosen to be investigated using the D-optimal design.

### 2.3. Thermodynamic Stability Tests

The thermodynamic stability study was conducted to assess the NE with an extended shelf life compared to standard emulsions. The standard emulsions are an energetically stable system and are more prone to being physically separated [20]. This is attributed to both the prevented coalescence brought on by the decrease in the interfacial energy as well as the advantageous adsorption of surfactant and cosurfactant occurring at the oil/water (o/w) interface [23]. Consequently, this investigation evaluated the thermodynamic stability of the prepared NE by subjecting it to centrifugation, consecutive freeze–thaw cycles, and heating and cooling cycles, respectively. The findings showed that all formulas completed the tests without signs of precipitation, phase separation, creaming, or cracking, demonstrating the stability of the developed NE.

### 2.4. Experimental Statistical Analysis

For the optimization and evaluation of the impacts of the two independent variables, namely oil concentration (A) and Tween 20 concentration in the Smix (B), on the three dependent variables, namely particle size (PS) (Y_1_), zeta potential (ZP) (Y_2_), and dissolution efficiency (DE%) (Y_3_), the D-optimal design was employed (Table 1). The selection of oil and Smix concentration ranges for the design was based on the construction of the ternary phase diagram, which was used to identify the NE region and ensure the formation of stable formulations. The D-optimal design was selected due to its efficiency for optimizing complex formulations by using mathematical algorithms with a limited number of experimental runs [23]. ANOVA was then used to analyze the data using the Design Expert^®^ program, version 13. The statistical model fit parameters for the optimization process are presented in Table S1. Table 2 shows the twelve experiments that resulted from the performed design.

#### 2.4.1. Variables Influence on the PS (Y_1_)

**Figure 2:**
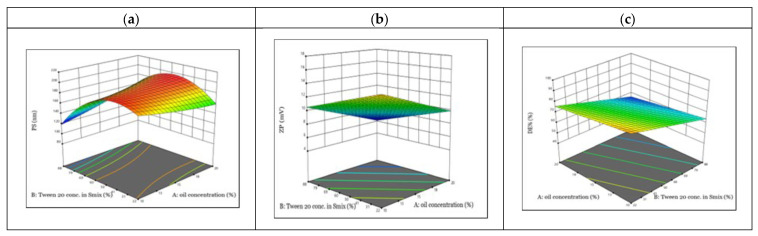
Three-dimensional response surface plots for the impact of factors A and B on (**a**) PS, (**b**) ZP (expressed as absolute value), and (**c**) DE%.

PS has a significant influence on the topical delivery of NE. A reduction in droplet size can result in effective diffusion across the stratum corneum [22]. The PS of the NE formulas that were prepared in this investigation varied from 123.10 ± 1.91 nm (F6) to 195.30 ± 3.31 nm (F8) (Table 2). It was evident from the design that increasing the oil concentration (A) resulted in an increase in the PS (Figure 2a). That might be because the droplets’ globules were getting bigger [20]. Furthermore, this might be attributable to an increase in the dispersion phase with a concurrent drop in the Smix portion [21,24]. On the other hand, increasing the concentration of Tween 20 in Smix (B) while keeping the oil content the same led to a considerable reduction in the formulation PS (Figure 2a). This was due to the fact that increasing the surfactant concentration might result in a larger amount of surfactant available for adsorption and development of a stable, densely packed surfactant film at the o/w interface that would reduce the size of the droplets [20]. Moreover, the prepared TRB-loaded NE showed polydispersity index (PDI) values ranging from 0.41 ± 0.025 (F11) to 0.72 ± 0.055 (F6) (Table S2).

#### 2.4.2. Variables Influence on the ZP (Y_2_)

ZP is a surface charge function whose value is associated with the dispersion systems’ physical stability [25]. All examined formulas had negative ZP values ranging from 6.60 ± 1.37 mV (F8) to 15.90 ± 0.77 mV (F9) (Table 2). The low ZP values could be due to the structure of the drug, containing a tertiary amine group that may shift values to a neutral sign. Negative surface charges of NE refer to an electrostatic repulsion that guarantees the formation of well-dispersed NE systems without coalescence [20]. The ZP values were presented in the design as absolute values to facilitate optimization across different formulations, regardless of charge direction. The design clearly shows that higher oil concentration has a negative impact on NE’s ZP absolute value (Figure 2b). This impact was attributable to the considerable enlargement in the size of the droplets upon raising the oil content. This might decrease the total charge distributed on the NE droplets [20]. Nevertheless, when the concentration of Tween 20 rose, the absolute values of ZP decreased significantly (Figure 2b). Raising the surfactant concentration above a precise level might promote the sudden elimination of OH-groups from the o/w interface, reducing the surface potential and hence the ZP. Although the prepared NE had relatively low ZP values (less than 30 mV), attributable to the large polyoxyethylene head groups of adsorbed Tween molecules, the prepared NE showed good stability. This was due to the steric repulsion that opposed the aggregation and stabilized the oil droplets in the formulations, accounting for the enhanced stability [24,26].

#### 2.4.3. Variables Influence on the DE% (Y_3_)

The evaluation of dissolution efficiency is an essential step for the detection of drug penetration into the skin. During the process of biological permeation, the drug should first be dissolved and released from the vehicles before being partitioned into the skin [25]. The examined NE formulas demonstrated remarkably high TRB release (Figures S1 and S2), with DE% values ranging from 55.16 ± 1.33% (F5) to 88.50 ± 1.84% (F9) (Table 2). We found that an increase in the oil content led to a decrease in the dissolution rate of TRB-loaded NE formulas and subsequently a reduction in their DE% values (Figure 2c). This might be due to the probable production of larger-sized NE droplets because of rising oil concentrations with decreased surface area accessible to the medium, therefore inducing a lesser proportion of dissolved drug [27]. Additionally, we found that the increase in Tween 20 concentration in the Smix negatively affected the DE% (Figure 2c). This might be due to the reduction in the ethanol concentration in the Smix by increasing the Tween 20 concentration, leading to an increase in the surface tension. When the cosurfactant concentration increases in the NE systems, this leads to reduced interfacial tension and increases the fluidity of the interface [25]. Moreover, the cosurfactant could improve the mobility of the oil’s hydrocarbon tail, allowing for their better penetration, hence boosting the nanoemulsification effectiveness [25]. Furthermore, the higher penetration of the cosurfactant into the surfactant monolayer interface could strengthen the nanoemulsification of the system, raising the cumulative percentage of drug release [28].

### 2.5. Optimization and Validation of Variables

The optimum TRB-loaded NE formula was chosen using Design-Expert^®^ software, considering the desirability criteria. The optimization goals were to minimize PS (Y_1_) and maximize both ZP (Y_2_) and DE% (Y_3_) (Table 1). The software’s proposed optimal formula (F7), which exhibited maximum desirability (0.728), was achieved by producing the TRB-loaded NE using peppermint oil, Tween 20, and ethanol with percentages of 10%, 22%, and 68%, respectively. The practical and predicted values had a satisfactory correlation (Table 3) with acceptable prediction error percentages (less than 10%) [29].

### 2.6. In Vitro Characterization of the Optimum TRB-Loaded NE Formula

#### 2.6.1. In Vitro Cumulative Release Study of TRB-Loaded NE Formula

**Figure 3:**
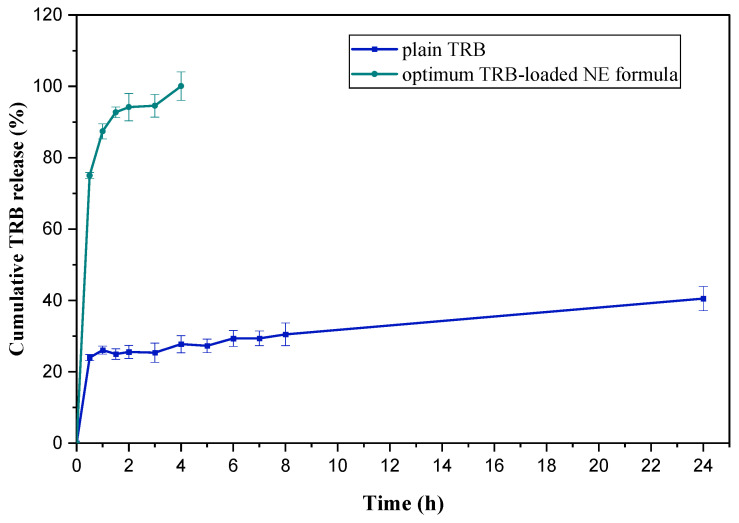
In vitro cumulative release profiles of the optimum TRB-loaded NE formula and plain TRB.

The in vitro release pattern of plain TRB and the optimum TRB-loaded NE formula (F7) was assessed using the dialysis bag method. As demonstrated in Figure 3, after 24 h, only about 40.00 ± 3.40% of a plain aqueous suspension of TRB was released owing to its minimal water solubility. This was proven by drug precipitation in the dialysis bag enclosing the plain TRB suspension. Nevertheless, the optimum TRB-loaded NE formula demonstrated a statistically significant increase in drug release rate (*p* < 0.05), as it reached 100% of the drug released within 4 h. In comparison, only about 27.00 ± 2.40% of TRB was released from the drug suspension at the same time. This was attributed to the small globule size of the optimized formula with consecutive increases in surface area available for the release media. Additionally, the higher polarity of the optimum formula (F7) by proper balancing between the oil and Smix led to excellent solubilization capability of NE for TRB [21].

#### 2.6.2. FT-IR Spectroscopy

**Figure 4:**
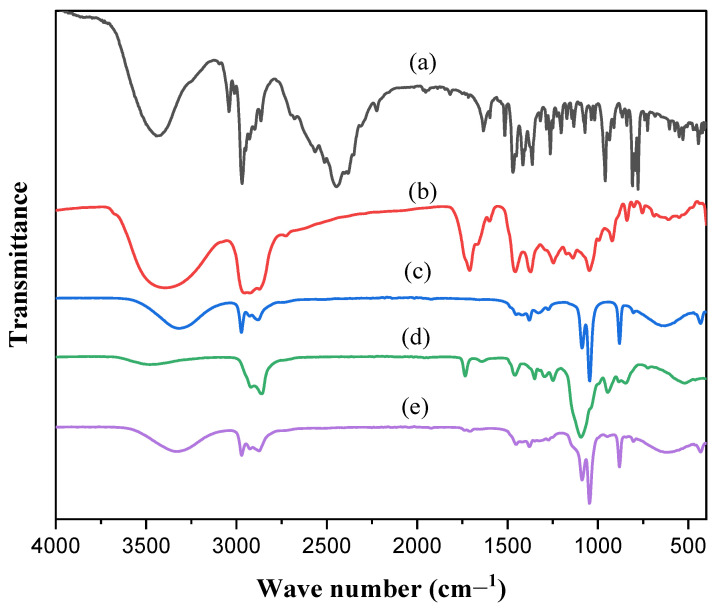
FT-IR of (**a**) plain TRB, (**b**) peppermint oil, (**c**) ethanol, (**d**) Tween 20, and (**e**) optimum TRB-loaded NE formula.

The FT-IR spectra of plain TRB, NE constituents, and the optimum TRB-loaded NE formula are illustrated in Figure 4. The FT-IR plain TRB (Figure 4a) exhibited characteristic peaks at 1408 cm^−1^ and 1384 cm^−1^ corresponding to the C-N and CH3 groups, respectively [6]. The peak at 3043 cm^−1^ was indicative of aromatic C-H groups, whereas the peak at 2983 cm^−1^ was ascribed to aliphatic C-H group stretching. Additionally, the TRB spectrum has a prominent peak of about 3400 cm^−1^ corresponding to OH and NH functional groups [30]. The FT-IR of peppermint oil (Figure 4b) displayed distinctive speaks at 3395 cm^−1^, 2955 cm^−1^, 2872 cm^−1^, 2726 cm^−1^, and 1709 cm^−1^, representing the O–H stretching band, C–H stretching band, aldehydic H–C=O, and C=O stretching band, respectively [20]. The FT-IR of ethanol (Figure 4c) gives a peak at 2981 cm^−1^ for C–H stretching vibrations. The peaks fall within the range of 1050 cm^−1^ to 1075 cm^−1^, representing the C–O stretching band for primary alcohols, while the peaks fall within the range of 1350 cm^−1^ to 1260 cm^−1^, representing the O–H bending band for primary alcohols. FTIR spectrum of Tween 20 (Figure 4d) demonstrated a broad peak at 3476 cm^−1^ corresponding to hydrogen-bonded O–H stretching. A double peak at 2920–2860 cm^−1^ represented asymmetric and symmetric methylene stretching vibrations. A sharp peak of stretch vibration of CH2–O–CH2 group was observed at 1095 cm^−1^. The peaks around 1734–1640 cm^−1^ originated from C=O groups. The FTIR spectrum of the optimum TRB-loaded NE (Figure 4e) showed that the distinctive groups of the NE components formed intermolecular hydrogen bonding with the O-H group of ethanol, as it was the bulk constituent included in the NE formula. These findings suggested that TRB might be uniformly mixed and incorporated into the NE components without any chemical interactions [31]. Furthermore, it outlined the ability of peppermint oil, Tween 20, and ethanol to incorporate the TRB characteristic functional groups into the NE formulation, explaining the significant improvement of TRB solubility [21].

#### 2.6.3. DSC Study

**Figure 5:**
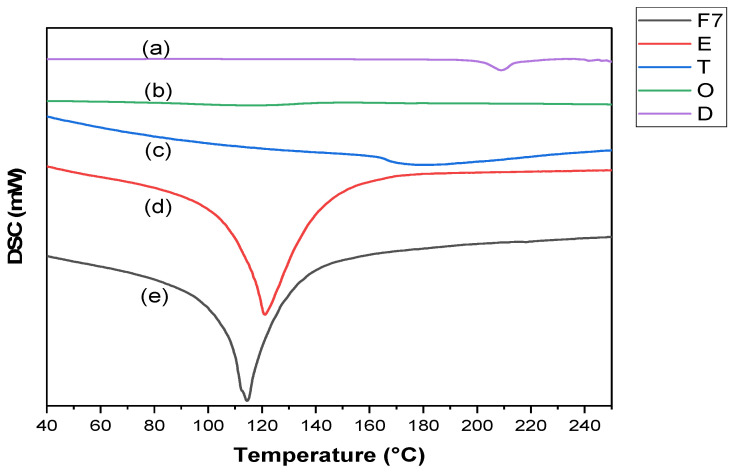
DSC curves of (**a**) plain TRB, (**b**) peppermint oil, (**c**) Tween 20, (**d**) ethanol, and (**e**) optimum TRB-loaded NE formula.

The DSC analysis was undertaken to study the possible interactions between the drug and excipients of the NE formula. The DSC thermograms of plain TRB, each NE constituent, and the optimum TRB-loaded NE formula are shown in Figure 5. Plain TRB’s DSC thermogram demonstrated a prominent endothermic peak at 215.50 °C. This peak represented the drug’s melting point and indicated its crystalline nature. Peppermint oil, Tween 20, and ethanol exhibited endothermic peaks as found in their DSC thermograms at 114.80 °C, 174.39 °C, and 121.89 °C, respectively. Interestingly, the DSC thermogram of the optimum NE formula did not exhibit the characteristic endothermic peak of plain TRB. This would indicate the molecular dispersion of the drug in the NE components, leading to an increase in the drug solubilization in the optimum NE formulation [21].

#### 2.6.4. TEM Study

**Figure 6:**
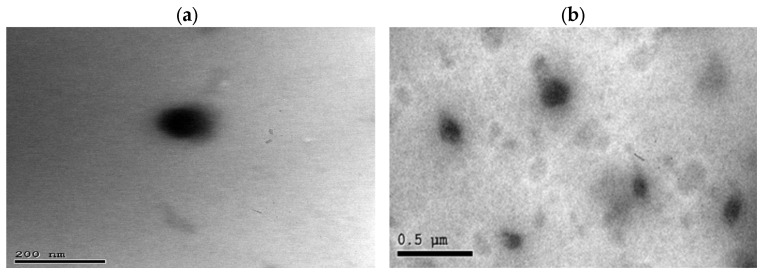
TEM images of the optimum TRB-loaded NE formula at different scales: (**a**) 200 nm and (**b**) 0.5 μm.

By investigating the TEM images, the surface morphology of the optimum TRB-loaded NE formula might be evaluated (Figure 6). The TEM pictures of the formula revealed dark-appearing spherical droplets in contrast to the brilliant surrounding media. The characteristic droplets’ spherical form would indicate their capability to facilitate the penetration of drug-loaded systems via the skin’s narrow pores [32].

### 2.7. Preparation of TRB-Loaded NEF Formulation

In our work, liquid foam was produced by simple mechanical shaking of a liquid solution that promoted foaming posteriorly, utilizing a non-propellant pump mechanism. Firstly, a blank foam was prepared separately, and then, the optimum NE formula was added to generate the NEF system. The main constituents used to prepare the foams were beneficial. The water-soluble polymer (HPMC-4000) could increase the viscosity of the foam, leading to improved foam stability and drug delivery [33]. Pluronic F127 could improve foam stability, increase foam viscosity, and enhance drug delivery through the skin [34]. Sodium dodecyl sulfate (SDS) could contribute to the formation and stabilization of the foam structure by reducing the surface tension of the liquid phase, allowing the formation of bubbles, and enhancing the dispersion of the active pharmaceutical ingredient within the foam matrix [35]. In addition, glycerin is a foam stabilizer [18], and PG as a humectant would help to retain moisture in the skin and improve the overall skin feel upon application.

### 2.8. Evaluation of the Prepared TER-Loaded NEF

Following the preparation of the foamable formula and the dispensation of the foam at the area of application, the product must retain its structural integrity for a specified duration [33]. The foam may be essentially absorbed by the skin, but it should not break down, collapse so quickly, or drop off the skin before the drug absorption [34]. The stability and structure of the generated foam should be assessed by characterizing its critical physical characteristics [19].

#### 2.8.1. Foam Calculated Parameters

The determination of foam parameters (FE, FLS, and FVS), which indicate the “before-application” stability, is critical for the pre-formulation production stage and the foam preparation acceptance [36]. Higher FE implies a more foamable formula, but higher FVS means maximal foam stability. Furthermore, FLS correlates with drain stability and has an inverse association with foam stability. The FE of the prepared TRB-loaded NEF formula was 125.00 ± 0.50%, showing a high foam stability [12]. The higher foam expansion was due to the utilization of the triplicate surfactant mixture (SDS, Pluronic F127, and Tween 20), which led to enhancing FVS and reducing FLS. This finding was in accordance with Eita A. et al. (2022) [12]. Although the prepared formula displayed a good FE, it is significantly lower than that of the blank foam, which showed an FE of about 350.00 ± 3.50%. This reduction was attributed to the presence of ethanol in the NE formulation that acted as a defoaming agent in the foam preparation, resulting in the destabilization of the foam and decreasing the foam stability and expansion but within an acceptable limit [37]. Moreover, ethanol solvent could dissolve the surfactant molecules, reducing their concentration at the air/liquid interface and leading to a decrease in the surface tension of the foam. This decrease in the surface tension could cause the foam to collapse more quickly [38].

#### 2.8.2. Foam Half-Life

The foam half-life is defined as the interval in which the volume of the foam decreases to half in comparison to its starting volume. The foam half-life was measured to assess the foam efficiency by demonstrating foam stability [19]. The prepared TRB-loaded NEF formula showed a half-life of about 90.00 ± 1.50 min. This long half-life was achieved by using high concentrations of surfactants (2% *w*/*w* and 4% *w*/*w* of SDS and Pluronic F127, respectively) [12]. The blank foam showed a half-life of 180.00 ± 2.50 min. The difference in the foam half-life between the blank foam and the prepared TRB-loaded NEF formula was due to the presence of Tween 20 in the NE formula. The observed decrease in the half-life might be attributed to the synergistic impact of non-ionic surfactants (Tween 20 and Pluronic F127), causing the maximum stability concentration to be exceeded [12]. Previous research has shown that when surfactant concentrations increase, the half-life also increases to a certain level, known as the maximum stability concentration. This was because exceeding the critical micelle concentration would minimize the surface tension and eventually produce micelles of diverse forms until producing a lamellar form of greater thermodynamic stability [39].

#### 2.8.3. Viscosity

The foam preparation process is significantly impacted by viscosity, which can affect the foamability and foam stability. Prolonging the lifespan of foams may be achieved by incorporating a low concentration of viscosity-increasing polymers into the liquid phase [18]. Gennari et al. (2019) observed that a viscosity-modifying agent was necessary to produce a foam with optimal qualities, where the foam development was infeasible with a concentration greater than 2% *w*/*w* [40]. The prepared TRB-loaded NEF formula displayed a viscosity of about 349.00 ± 1.75 cP, while the blank foam displayed 458.00 ± 3.75 cP. This high viscosity was attributed to the high HPMC concentration (2% *w*/*w*). Therefore, it was necessary to promote the development of foam with acceptable texture and optimal spreading characteristics [41].

#### 2.8.4. Foam Collapse

**Figure 7:**
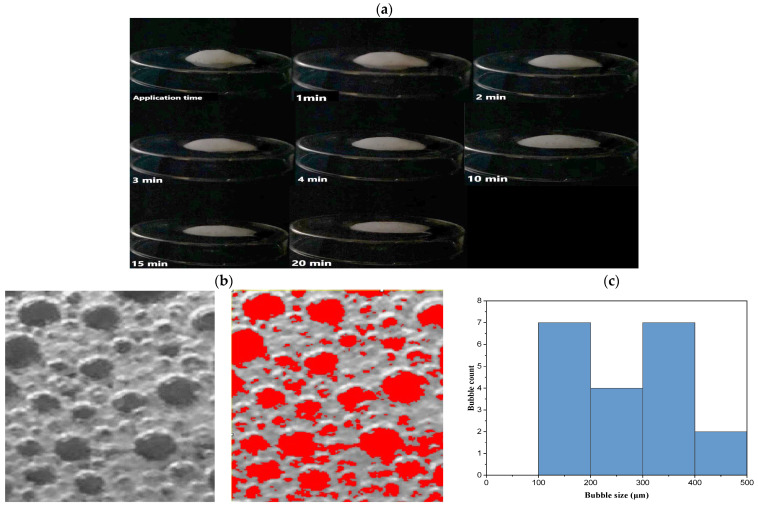
(**a**) After actuation, foam collapse time was shown from the side view, demonstrating a stable structure with a lengthy collapse time. (**b**) The size and distribution of the foam’s bubbles were assessed by ImageJ software, version 1.53e, Scale bar = 50 µm. (**c**) The histogram was produced using ImageJ software.

Determining the foam collapse ability helps assess the foam stability. The lengthy collapse time implies strong foam’s thermal stability [19]. As demonstrated in Figure 7a, the foam collapse behavior at different time periods suggested excellent stability, particularly with longer collapse times. The produced foam belonged to the group of foams known as breakable foams that developed stable breaking properties by applying shear stress [17]. These breakable foams enable easy dermal application and optimal administration since they maintain stability for an appropriate time duration (20 min). Ideally, the foam construction should exhibit stability for a duration beyond 2–3 min [18].

#### 2.8.5. Bubble Size Estimation

As demonstrated in Figure 7b,c, foam bubble morphology was assessed using ImageJ software, which generated histograms confirming the formation of small, relatively uniform bubbles. The average bubble size, analyzed from multiple images (*n* = 3), ranged between 100 ± 5.6 µm and 500 ± 10.2 µm, indicating a narrow size distribution. This uniformity is positively correlated with foam stability and spreadability. Such morphological consistency plays a crucial role in foam performance, particularly affecting rheological properties, drug delivery uniformity, and user acceptability [12]. As noted by Eita A. et al. (2022), foam instability might result from the uneven distribution pattern. These small and uniformly distributed bubbles might result from the inclusion of a high SDS content [12].

#### 2.8.6. Measurement of PS, PDI, and ZP

**Figure 8:**
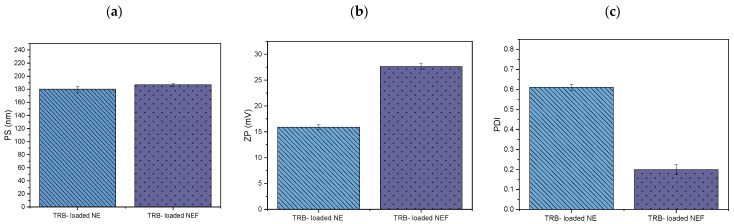
Comparison between the optimum TRB-loaded NE system and the TRB-loaded NEF regarding PS (**a**), ZP (expressed as absolute values) (**b**), and PDI (**c**), respectively.

Specific aspects were evaluated to find any variations to confirm the system’s suitability after incorporating the optimum formula of TRB-loaded NE in the foam system. According to Khan et al. (2022), the physicochemical characteristics of nanoparticles have an impact on topical drug delivery. Hence, the investigation was conducted to identify any significant changes in the PS, PDI, and ZP [42]. Following the data analysis, the illustration in Figure 8a–c demonstrated minor variations with insignificant effects on the PS, while the ZP and PDI showed significant variations. After loading the optimized NE formula into the foam system, a significant increase in the ZP values from −13.90 ± 0.45 mV to −27.60 ± 0.55 mV was observed, demonstrating the capability of the foam system to improve the stability of the produced formula. Moreover, the PDI of the optimized TRB-loaded NE significantly decreased from 0.61 ± 0.015 to 0.19 ± 0.025 after being incorporated into foam, reflecting the enhancement in the uniformity and the narrow PS distribution [25].

#### 2.8.7. In Vitro Cumulative Release Study of TRB-Loaded NEF Formula

**Figure 9:**
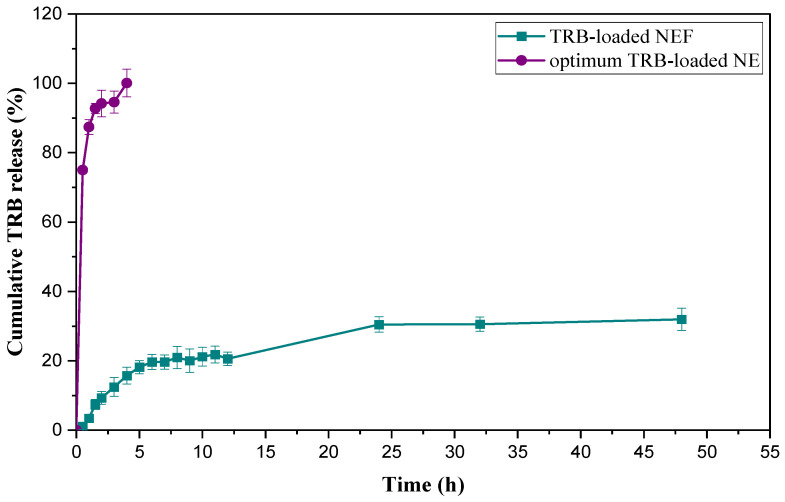
Cumulative release profiles of TRB-loaded NEF and the optimum TRB-loaded NE.

The release profiles of the optimum TRB-loaded NE (F7) and the TRB-loaded NEF formulas are graphically illustrated in Figure 9. The release rate of TRB from the TRB-loaded NEF formula was significantly reduced compared to the TRB-NE formula. The delayed release rate of the drug from the foam system could be ascribed to the coverage of the drug-containing oil droplets by a layer of polymer in the foam formulation. Therefore, the drug was required to transit an extended path before it reached the release medium, leading to a reduced drug release in a sustained manner [20]. Moreover, in terms of foam components, as described by Zhao et al. (2010), surfactants such as Pluronic P127 would improve the solubility of the drug while decreasing its release rate [41].

#### 2.8.8. Ex Vivo Permeation Study

**Figure 10:**
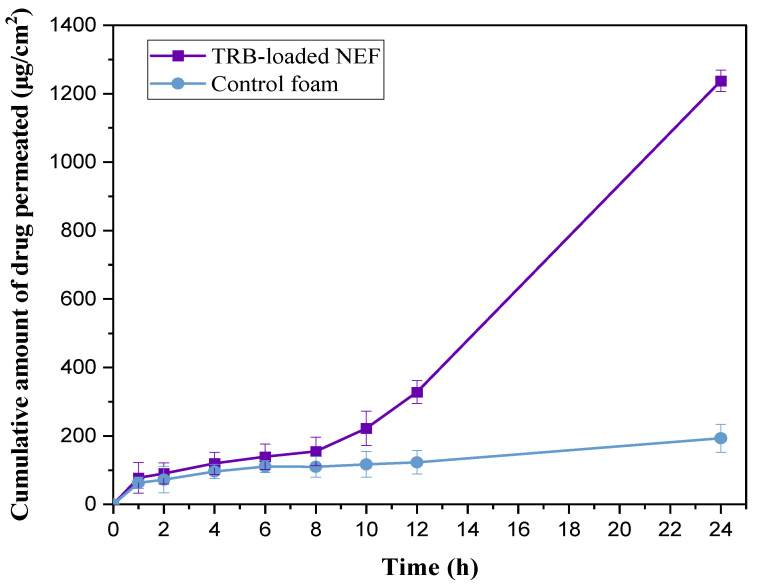
Ex vivo permeation profiles of TRB-loaded NEF and TRB-loaded control foam.

The ex vivo permeation of TRB through the abdominal skin of a rabbit from TRB-loaded NEF or TRB-loaded control foam was assessed as a predictor of the actual in vivo behavior. As seen in Figure 10, the skin permeability of TRB was greatly enhanced by incorporating the optimal TRB-loaded NE formula into the foam system compared to the TRB-loaded control foam (*p* < 0.05). The cumulative amounts of drug penetrated via the skin from TRB-loaded NEF and TRB-loaded control foam were 1237.11 ± 44.33 μg/cm^2^ and 193.34 ± 34.45 μg/cm^2^, respectively, at 24 h. The enhanced permeability of NEF loaded with TRB might be ascribed to two factors. First, the nanosized particles in the NE formulation aided in solubilizing the drug and increasing the surface area for drug penetration. Secondly, the capability of the components in the formulation to permeate the lipid barrier of the skin’s surface was observed [43]. As reported before, Tween 20 could improve drug permeation, as it could permeate the spaces between cells in the outermost layer of the skin, increasing flexibility and finally dissolving and removing lipid components. This is succeeded by interaction and attachment to keratin filaments, causing a disturbance inside the corneocyte [43]. Furthermore, ethanol used in the NE formulation was recognized for its effectiveness and safety in promoting drug permeation across biological membranes by modifying the solubility characteristics of the tissue, hence enhancing drug partitioning into the membrane [44]. Notably, in addition to the peppermint oil solubilizing capacity for TRB, it possesses penetration-enhancing and anti-inflammatory properties that can synergistically support its anticancer activity [45].

The calculated permeation parameters of the two studied formulas are provided in Table 4. The Jss of TRB from the NEF formulation was 47.53 ± 4.98 µg/cm^2^·h^−1^, which was significantly greater than that of the TRB-loaded control foam (*p* < 0.05). In addition, the enhancement of drug penetration from TRB-loaded NEF was 7.43 times more than that of TRB-loaded control foam. These findings supported the potential of NEF formulation in enhancing the skin’s ability to permeate the loaded drug deeply through the skin layers. The lag time (Tlag) of TRB-loaded NEF was calculated as 1.91 ± 0.37 h, which was notably a short time, indicating faster onset of permeation. Markedly, rabbit skin is typically more permeable than human skin, and therefore, the absolute flux values reported may overestimate actual human skin permeation. However, as the same skin model was consistently used across all tested formulations, the comparative enhancement in flux remains meaningful for evaluating formulation performance during this developmental phase.

#### 2.8.9. Histopathological Study

**Figure 11:**
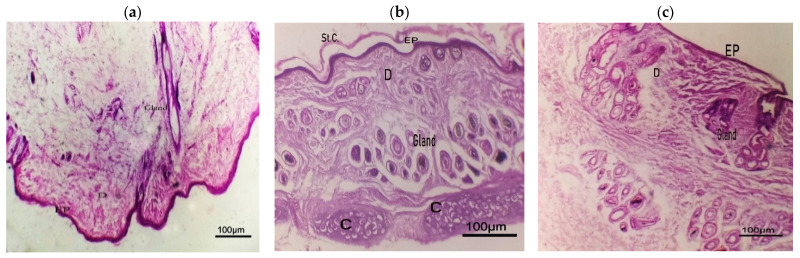
Histopathological study of rabbit skin samples treated with (**a**) normal saline, (**b**) TRB-loaded control foam, and (**c**) TRB-loaded NEF. Abbreviations: StC, stratum corneum; C, cartilage; EP, epidermis; D, dermis.

Histopathological investigations were performed to evaluate the safety of the topically applied NEF formulation. A comparison was made between rabbit skin samples treated with TRB-loaded NEF or TRB-loaded control foam formulas and a normal skin sample treated with normal saline (Figure 11). Normal skin had a distinct anatomical composition consisting of epidermis, dermis, and subcutaneous strata, with typical skin appendages and vascular systems (Figure 11a). Interestingly, no evidence of inflammation and/or irritation was found in the dyed skin samples treated with either TRB-loaded control foam or TRB-loaded NEF formula, as shown in Figure 11b and Figure 11c, respectively. Moreover, no changes in skin structure were seen after topical administration of the studied formulas. These findings would clearly demonstrate the safety and tolerability of the drug-loaded NEF formula [20].

### 2.9. Cytotoxicity Assessment

#### 2.9.1. Cell Viability Assay

**Figure 12:**
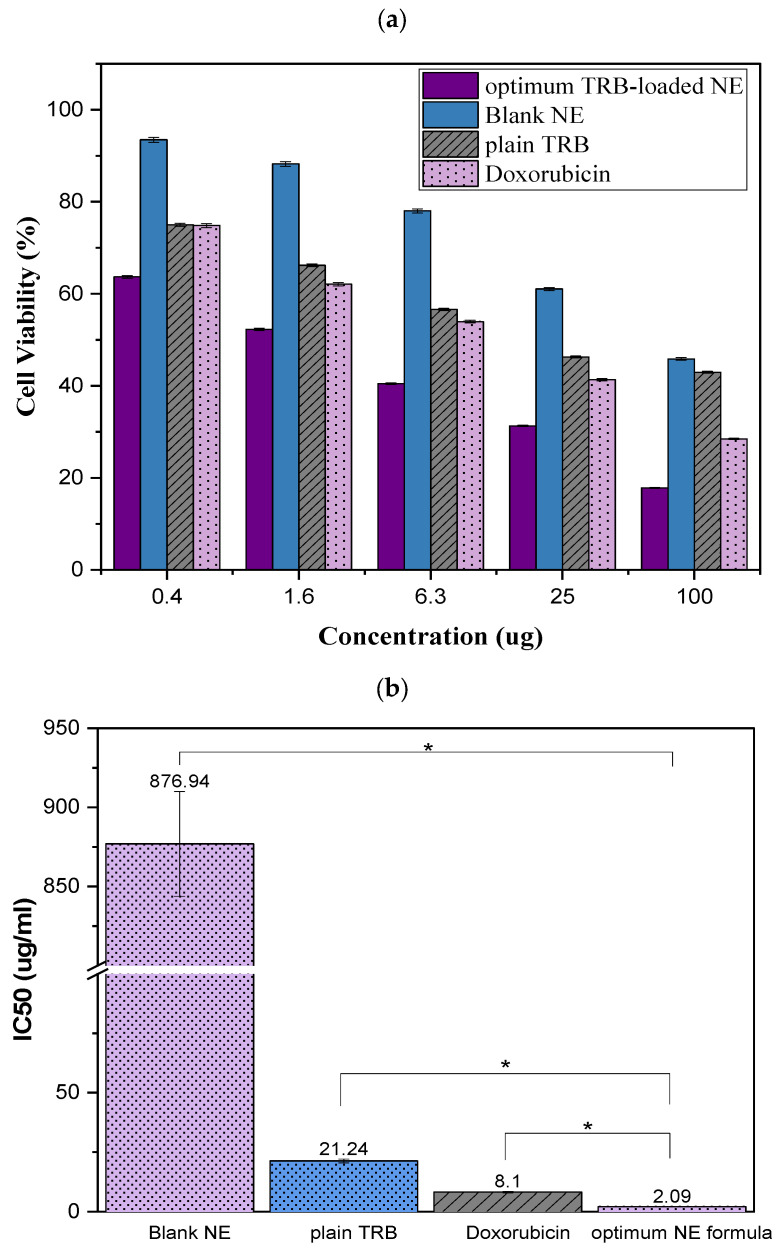
(**a**) Cell viability on A-431 cancer cells; (**b**) IC50 of the optimum TRB-loaded NE, blank NE, plain TRB, and doxorubicin on A-431 cell line. * significant at *p* < 0.05.

To assess the anti-cancer potential of the repurposed antifungal TRB, its cytotoxic efficacy against tumor tissue was evaluated [11]. We assessed the cytotoxicity of plain aqueous suspension of TRB, the optimum TRB-loaded NE formula, and the blank NE (peppermint oil, Tween 20, and ethanol) with the MTT assay using doxorubicin as a positive control in human epithelioid squamous carcinoma cell line (A-431). The untreated A-431 cells (cells without exposure to drug or vehicle) in the MTT assay served as the negative control, representing 100% cell viability. Notably, no prior reports have demonstrated a TRB-based nanoformulation with such a cytotoxic profile against the tested skin cancer cell lines, underscoring the novelty and translational promise of our approach. After 48 h of treatment with various concentrations of the studied samples, the A-431 cell line’s cell viability declined in a dose-dependent manner (Figure 12a). As observed in Figure 12b, the growth inhibitory concentration (IC50) values were used to quantify the cell viability. The IC_50_ values were calculated based on cell viability relative to the untreated control group. The IC50 of the plain TRB was found to be 21.24 ± 0.80 μg/mL, showing the drug has intermediate anti-cancer activity against skin cancer. On the other hand, the IC50 value of the optimal formula was significantly lowered (*p* < 0.05) to 2.09 ± 0.08 μg/mL compared to the plain drug. Conversely, the IC50 value of the blank formula increased significantly (*p* < 0.05) to 876.94 ± 33.10 μg/mL compared to the other formulas. Surprisingly, the optimum formula also displayed a significant decrease in the IC50 compared to doxorubicin. We found that the optimal formulation of TRB-loaded NE increased the cytotoxicity of TRB by about 10 times against the A-431 cell line when compared to plain TRB. This was due to the small droplet size and high surface area of NE, which allowed enhancing TRB delivery and bioavailability at the cellular level. These findings suggest that the NE carrier improves intracellular uptake and retention of TRB, leading to more effective inhibition of A-431 skin cancer cell viability [9].

#### 2.9.2. Apoptosis Assay

**Figure 13:**
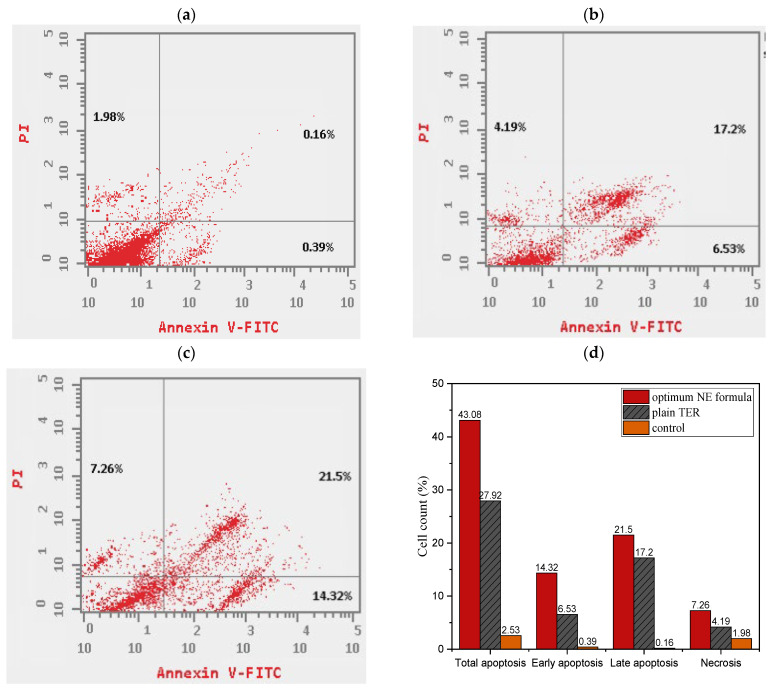
(**a**–**c**) Apoptosis study using flow cytometry for untreated cells, plain TRB, and optimum TRB-loaded NE, respectively. (**d**) Cell counts percentage analysis.

To study the TRB potential mechanism of cytotoxicity in the A-431 cell line, we utilized flow cytometry to measure cell apoptosis using an Annexin V/PI test kit. The test evaluated early apoptosis (Q4), late apoptosis (Q2), necrosis (Q1), and cell viability (Q3), as seen in Figure 13a–c. The experiments consisted of untreated A-431 cell lines (serving as controls) in addition to cells treated with both plain aqueous suspension of TRB and the optimum TRB-loaded NE formula. Figure 13d demonstrates that the two treatments markedly elevated total, early, and late apoptosis compared to the control cells (*p* < 0.05). This finding was correlated with previous investigations on the anti-tumor properties of TRB [14]. Cytotoxicity can be seen in both plain TRB and the optimal formula of TRB-loaded NE, mainly owing to the combined impact of early and late apoptosis, while necrosis had little impact in comparison. The investigation showed that plain TRB increased cellular apoptosis by 11.04-fold compared to the control. Nevertheless, apoptosis in A-431 cells treated with the optimum formula surged by about 17.03-fold compared to the control. Furthermore, the proportion of overall apoptotic cells in the optimized formula was about 1.54 times greater than in the plain TRB. These findings proved the NE’s ability to induce programmable cell death in the skin cancerous cells [9].

#### 2.9.3. Cell Cycle Study

**Figure 14:**
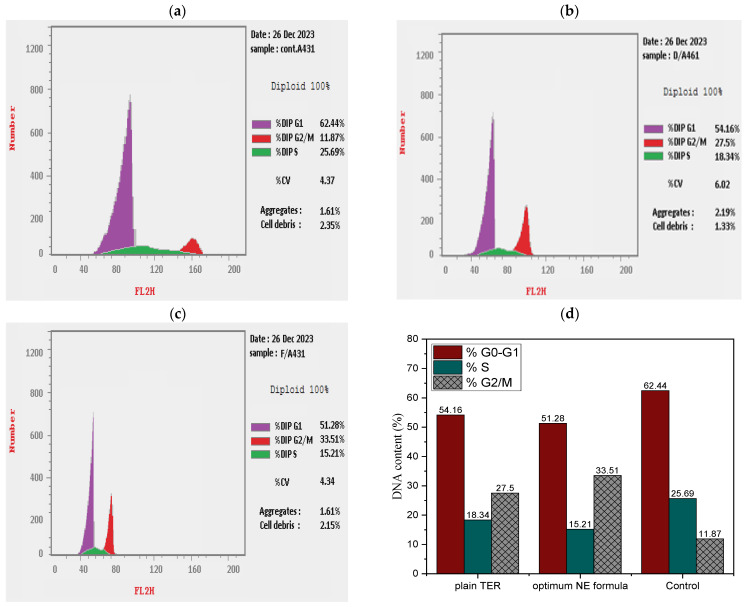
(**a**–**c**) Cell cycle study employing flow cytometry for untreated A-431 cells, plain TRB, and optimal TRB-loaded NE formula, respectively. (**d**) Percentage of cell cycle analysis.

The cell cycle is a fundamental biological process that encompasses DNA replication, nuclear division, and cytoplasm partitioning for cell reproduction [46,47]. The cell cycle behavior of A-431 cells after treatment by plain aqueous suspension of TRB and the optimum TRB-loaded NE formula is depicted in Figure 14a–c, utilizing flow cytometry and nuclear PI staining. We found that the plain TRB and the optimum NE formula caused high cell accumulation in the G0–G1 phase with 54.16% and 51.28% of cells, respectively. They also increased the number of cells in the G2/M phase by 2.32 and 2.82 times, respectively, when compared to the control (Figure 14d). In addition, the S phase of cells treated with plain TRB and the optimum formula exhibited respective reductions of 1.40-fold and 1.69-fold compared to the control. The studies reported here were entirely consistent with those reported by Huang C. et al. (2008) [14].

### 2.10. In Silico Study of TRB-Loaded NEF

**Figure 15:**
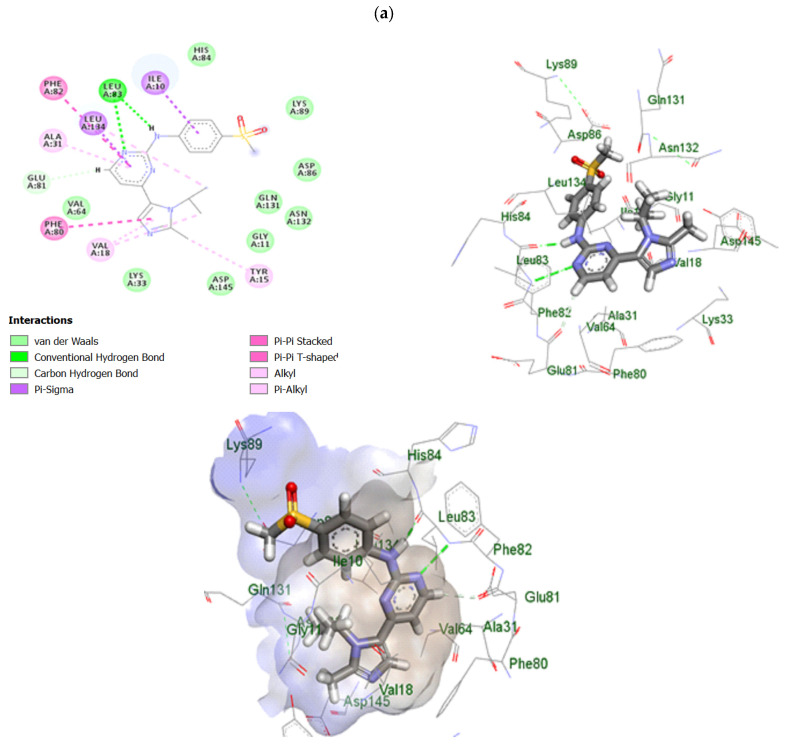

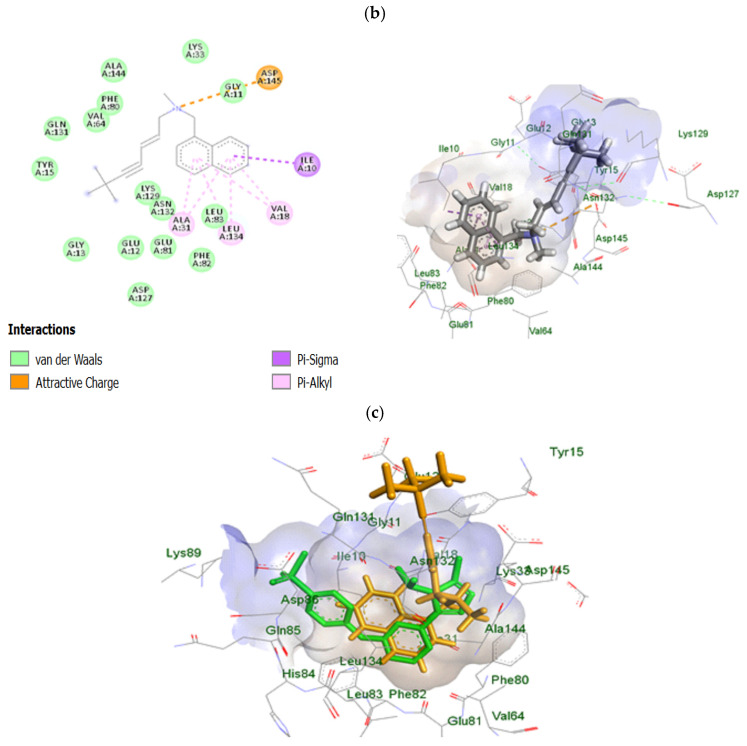
Two- and three-dimensional interaction diagrams of (**a**) reference compound AZD5438, (**b**) TRB under investigation, and (**c**) molecular overlay of both drugs in the active site of CDK 2.

TRB was reported to be an anti-proliferative agent in different cancer types, such as oral [48], colon [49], and liver [50]. The cell cycle was arrested by inhibition of CDK 2, 4 [48], AMPK-mTOR axis [50], and Raf-MEK-ERK axis. Thus, docking studies were carried out against CDK 2, as it was reportedly inhibited in squamous cell carcinoma [48]. The binding scores of TRB were very promising compared to the reference drug (AZD5438), which acts as an ATP-competitive inhibitor. We chose AZD5438 as a reference compound due to its well-documented activity as a CDK2 inhibitor, which reinforces the relevance of targeting this protein in cancer proliferation pathways. Docking studies of TRB against the active site of CDK 2 resulted in a binding score of −7.22 Kcal/mol compared to AZD5438 scoring −8.93 Kcal/mol. The RMSD between the docked pose and the crystallographic conformation was 0.85 Å, indicating reliable reproduction of the native binding mode, as RMSD ≤ 2.0 Å is considered acceptable for successful redocking. It interacted with the reported key amino acids, such as Ile10, Tyr15, Lys33, Val64, and Leu83 [51]. TRB occupied the active site compared to the reference drug, where the naphthalene ring resided in the hydrophobic pocket formed by Ile10, Val18, Ala81, and Leu134, as depicted in Figure 15. The docking results supported the observed antiproliferative activity of TRB, reinforcing its inhibitory activity.

## 3. Materials and Methods

### 3.1. Materials

TRB was a thoughtful gift from the Egyptian Pharmaceutical Industries Group (Obour, Egypt). Oleic acid and peppermint, grape seed, lemon, eucalyptus, lavender, and frankincense oils were purchased from Nefertari Natural Body Care Line (Cairo, Egypt). Polyethylene glycol 400 (PEG 400), sodium dodecyl sulfate (SDS), Pluronic F127, span 80, hydroxypropyl methylcellulose 4000 (HPMC 4000), and cellulose membranes (molecular weight cut off 12,000) were obtained from Sigma-Aldrich Chemical Co. (St. Louis, MO, USA). Transcutol P was received from Gattefossé Co. (Saint-Priest, France). Disodium hydrogen orthophosphate, potassium dihydrogen orthophosphate, absolute ethanol (99%), glycerin, propylene glycol (PG), Tween 20, and Tween 80 were purchased from El-Nasr Pharmaceutical Chemicals Company (Cairo, Egypt). Each chemical and reagent was of analytical grade.

### 3.2. Methods

#### 3.2.1. Solubility Study of TRB in the NE Formulation Constituents

In sealed glass vials, excessive quantities of TRB were combined with 3 mL of different oils, surfactants, and co-surfactants. The vials were then shaken in a thermostatic shaking water bath (Lab-Line Instruments, Melrose Park, IL, USA) for 48 h at 37 ± 1 °C. The specimens were centrifuged at 15,000 rpm for 15 min at 25 ± 1 °C. At λmax 283 nm, the TRB concentration in the filtrates was determined using an ultraviolet–visible (UV–vis) spectrophotometer (Shimadzu UV 1650 Spectrophotometer, Kyoto, Japan). The investigated oils were oleic acid, peppermint, grape seed, eucalyptus, lavender, frankincense, and lemon oils. As surfactants, Span 80, Tween 20, and Tween 80 were tested, and as co-surfactants, ethanol, PG, PEG 400, and Transcutol P were studied.

#### 3.2.2. Ternary Phase Diagram Construction

The selection of oil, surfactant, and co-surfactant for NE preparation was based on their potential to increase the solubility of the drug within these components [52]. The ternary phase diagram was developed to detect the concentration of each component of NE that demonstrates the highest solubilization of TRB. The chosen oil was plotted against the surfactant and co-surfactant using the AUTOCAD^®^ software, version 22. The phase diagram contained a group of points that were generated and examined by visual observation to produce clear solutions after dilution with a constant volume of distilled water (1:100), stirred by a magnetic stirrer (Model MSH-20D, Witeg Labortechnik GmbH, Wertheim, Germany) at 200 rpm for 15 min at 25 ± 1 °C [20]. After water dilution, the area exhibiting a rapid and transparent transformation was designated as a self-NE-containing area. It was speculated that the bluish-white points represented self-microemulsion formulations, whereas the milky-turbid points was assumed to contain macroemulsion formulations. The formulas with self-NE characteristics were selected for further investigation.

#### 3.2.3. Experimental Design of TRB-Loaded NE

Using the D-optimal design (Design-Expert^®^ software, version 13), the effect of the independent variables, oil concentration (A), and Tween 20 concentration in the surfactant/co-surfactant mixture (Smix) (B) on the dependent variables PS (Y_1_), ZP (Y_2_), and DE% after 24 h (Y_3_) was investigated. The target goals of the studied responses and the lower and upper levels of the factors are shown in Table 1. Table 2 shows the twelve experiments that resulted from the performed design.

#### 3.2.4. Optimization of Formulation Components

Statistical analysis via one-way analysis of variance (ANOVA) was performed to evaluate the optimal fitness of the data. The relationships among the independent variable and the dependent responses were explored [53]. After that, the optimization technique was used to pick the optimized NE formula based on the goals shown in Table 1. To validate the chosen design, the practical values of the optimal NE formula were compared to those predicted by the program. The percent of prediction error was then computed using the equation below [54].

(1) $$Prediction\;error\;(\%)=\frac{predicted\;value-observed\;value}{predicted\;value}\times 100$$

#### 3.2.5. Preparation of TRB-Loaded NE

Utilizing the spontaneous emulsification method, TRB-loaded NE were prepared [20]. Firstly, the proper amounts of surfactant, co-surfactant, and oil were combined at the optimal proportions determined from the phase diagram investigations. Subsequently, a precise quantity of TRB (10 mg) was introduced. After that, the prepared formulations were vortexed for 10 min, resulting in the creation of the NE.

#### 3.2.6. Characterization of TRB-Loaded NE

##### Thermodynamic Stability Test

In this study, the developed NE formulations were subjected to three stages to evaluate their thermodynamic stability. In the first step, the tested samples were centrifuged at 15,000 rpm for 30 min at 25 ± 1 °C. The samples were then examined, while those that were separated, creamed, or cracked were discarded. In the second step, the samples were subjected to six successive heating cycles (45 °C) and cooling (4 °C) for at least 48 h at each temperature to assess how temperature changes affected the prepared NE’s stability. In the third step, the samples were freeze–thawed three times at temperatures ranging from −20 °C to 25 °C for 48 h each to test the NE’s efficient dispersibility.

##### Measurement of PS, ZP, and PDI

The PS was assessed using the dynamic light scattering (DLS) technique utilizing Zetasizer (Nano–ZS90, Malvern Instruments Ltd., Malvern, UK). Before the assessment, the investigated samples were suspended in distilled water, and an appropriate dilution (1:100) was achieved at 25 °C. They were inserted into a disposable glass zeta cell to determine the ZP of the examined samples. The findings of the tested formulas were then reported.

##### In Vitro Cumulative Release Study and DE% Measurement

The release behavior of the tested formulations was investigated by the dialysis bag approach, utilizing a thermo-balanced shaker water bath [6]. Briefly, a predetermined quantity of NE formulas (equivalent to 10 mg TRB) was placed into the dialysis bags and knotted at both ends. The prepared samples were then suspended in a 100 mL phosphate buffer (pH 5.5) as a receptor medium warmed to 37 ± 0.5 °C and agitated at 100 rpm in a shaking water bath equipped with a thermostat. Consistently, 3 mL of receptor medium was withdrawn, filtered using a 0.45 µm filter, and replaced with an equivalent volume of fresh medium, as the sink condition could be maintained. The amount of drug in the tested samples was then evaluated using UV spectrophotometry at a wavelength of 283 nm. The release data were expressed as the DE%, which is defined as the area under the dissolution curve as a percentage of the total area that would represent 100% dissolution over the same time. The mean DE% after 24 h values were used to examine the release profiles. These values were determined by employing the trapezoidal approach by utilizing the DDsolver add-in in Microsoft Excel software [20].

##### Fourier-Transform Infrared (FT-IR) Spectroscopy of the Optimum TRB-Loaded NE

The FT-IR examinations were performed using an FT-IR apparatus (8400 S, Shimadzu, Kyoto, Japan) using a KBr disc over the frequency range of 500–4000 cm^−1^ at a resolution of 4 cm^−1^.

##### Differential Scanning Calorimetry (DSC) of the Optimum TRB-Loaded NE

The DSC study was conducted utilizing DSC equipment coupled to a TA-501 thermal analyzer (DSC-50, Shimadzu, Japan). In a closed aluminum pan, the samples (5 mg) were heated at 10 °C/min with a nitrogen flow of 20 mL/min within a 25–250 °C temperature range.

##### Transmission Electron Microscopy (TEM) of the Optimum TRB-Loaded NE

The morphology of the tested sample was determined using the TEM technique. After diluting the formula, it was sonicated for 10 min and left to dry on a copper grid. Then, the dried sample was examined by the transmission electron microscope (JEM-2100, JEOL, Tokyo, Japan) at 80 kV.

#### 3.2.7. Preparation of TRB-Loaded NEF

In this investigation, a non-propellant foam system was prepared using the approach described by Eita et al. (2022) with some adjustments [12]. Initially, to prepare blank foam systems, 2% W/V HPMC was mixed with 0.5% W/V PG and 1.5% W/V glycerin. After that, 2% W/V SDS and 4% W/V Pluronic F127 were placed into the previous mixture and blended with 3 mL of distilled water using a magnetic stirrer for 10 min at 1500 rpm. Finally, 1 mL of the optimum TRB-loaded NE (containing 10 mg TRB) was added dropwise to the prepared foam and stirred until homogeneity was achieved. During the application process, foam production from the aqueous solution was accomplished using a non-propellant pump.

#### 3.2.8. Evaluation of the Prepared TRB-Loaded NEF

##### Foam Calculated Parameters

Various calculated factors were used in foam assessment to determine the foamability and stability features. To assess the foam stability, a foam solution was subjected to homogenization by high-speed stirring, leading to foam development on the solution’s surface. Subsequently, sequential photos of the foam were captured to determine the foam height in relation to time [12]. A particular amount of the aqueous solution (4 mL) was employed in foam generation and visually observed after 30 min. The volume of the starting solution and the developed foam as well as the volume of the foam and the resultant drain following the examined period were collected. The calculated parameters were foam expansion (FE), foam volume stability (FVS), and foam liquid stability (FLS), as illustrated in Equations (2)–(4) [18].

(2) $$FE\%=\frac{Vfm-Vft}{Vft}\times 100$$

(3) $$FVS\%=\frac{Vfm/30\;\min}{Vft}\times 100$$

(4) $$FLS\%=\frac{Vl/30\;\min}{Vft}\times 100$$

where Vfm is the resultant foam volume, Vft is the starting solution volume in mL, Vfm/30 min represents the foam volume in mL after 30 min, and Vl/30 min represents the drained volume in mL after 30 min.

##### Foam Half-Life

Foam stability may also be assessed by the foam’s half-life, which is the time needed to reach half of the original volume following foam generation. A longer half-life period suggests preferable stability [12].

##### Viscosity

The viscosity of the liquid formulation has a significant impact on its ability to generate foam when pumped. Viscosity was determined in triplicate using a Brookfield DV3T Viscometer (Brookfield Engineering Laboratories, Inc., Middleboro, MA, USA), spindle number 42, at a temperature of 25 ± 2 °C and a speed of 250 rpm.

##### Foam Collapse Capability

The capacity to estimate foam that would be present at the application site after a single pump from the propellant-free pump device onto a piece of glass is known as foam collapsing capability. Side-view images of the actuated foam were captured. The height was measured, and the collapse process was recorded at various time intervals.

##### Bubble Size Estimation

The image evaluation was carried out on a foam image obtained after the foam was actuated on a glass plate. The bubble size and distribution were determined using ImageJ software (version 1.53e, NIH Image, Bethesda, MD, USA). The program was used to edit the photographs, and the size distribution histogram was compared to the mean size of the globule bubble. The mean bubble size was calculated from multiple images (*n* = 3) to assess the uniformity and structural characteristics of the foam.

##### Measurement of PS, ZP, and PDI

The PS, ZP, and PDI of TRB-loaded NEF were assessed to validate foam stability. Additionally, it was critical to represent any alterations in the distinctive properties of the optimum NE formula following integration into the foam system. The measurement method is reported in “Section Measurement of PS, ZP, and PDI”.

##### In Vitro Cumulative Release Study of TRB-Loaded NEF Formula

Utilizing the dialysis bag approach, the percentage of the drug released from the prepared NEF formula was estimated, as mentioned in “Section In-Vitro Cumulative Release Study and DE% Measurement”. Definite amounts of the optimum TRB-loaded NE and TRB-loaded NEF equivalent to 10 mg of TRB were examined.

##### Ex Vivo Study

The animal breeding facility at Misr University for Science and Technology in Egypt provided three adult male rabbits weighing 1700–1900 g each. Before the experiments, the animals were kept at room temperature for a week in a 12 h light, 12 h dark cycle with free access to food and water. The studies were carried out in accordance with the recommendations of the Faculty of Pharmacy’s Institutional Animal Care and Use Committee (IACUC; PT/REC/34), approved on 2 April 2024.

In this investigation, the abdominal skin of a rabbit was used. First, the rabbit’s hair was gently removed using an electric clipper. The animals were then slaughtered, and their skins were removed. The adipose tissues were removed, and the skin samples were kept moist overnight in phosphate buffer (pH 5.5) at 4 °C [27].

The ex vivo penetration of the TRB-loaded NEF and control foam containing plain aqueous suspension of TRB was examined using fresh skin from the slaughtered rabbit. A particular weight of the formula (equal to 10 mg TRB) was introduced into a diffusion cell’s donor compartment. The receiver compartment held 100 mL of PBS (pH 5.5) and ethanol (70:30), as reported by Y. Yang et al. (2015), and sodium azide (0.02%) as a preservative [54,55]. At various time intervals, 3 mL samples were withdrawn and substituted with an equivalent volume of new release medium so that the sink condition could be maintained. The drug content was detected spectrophotometrically at 283 nm. The permeation parameters were calculated following the underlying equations [21].

(5) $$Jss=\frac{Slope\;of\;the\;linear\;part\;of\;graph}{area\;of\;diffusion\;cell}$$

(6) $$Kp=\frac{Jss}{Co}$$

(7) $$Er=\frac{Jss\;(formulation)}{Jss\;(control)}$$

where Jss is the steady-state drug flux, Co is the initial drug concentration, Kp is the permeability coefficient, and Er is the enhancement ratio.

##### Histopathological Study

The even-sized samples of the rabbit abdominal skin immersed in phosphate buffer (pH 5.5) were investigated. The first sample was an untreated skin piece (negative control). The second sample was treated with the TRB-loaded control foam, and the third sample was treated with the TRB-loaded NEF formula following the completion of the ex vivo permeability study. After the skin samples were removed from the permeation medium, they were rinsed with fresh medium for about one h and then preserved in 10% formalin for the night. Following this, the skin samples were vertically sliced, dried using ethanol, and embedded in paraffin bars. The tiny slices were then stained with hematoxylin and eosin. Finally, these stained slides were seen using a polarizing microscope (Radical Scientific Equipment Pvt. Ltd., Mumbai, India) with a digital camera (Nikon Optiphot, Nikon Corporation, Tokyo, Japan).

#### 3.2.9. Cytotoxicity Evaluation of TRB-Loaded NEF

##### Cell Culture

This investigation utilized a human epithelioid squamous carcinoma (A-431) cell line as a cancerous cell culture. These cells were obtained from the American Type Culture Collection (ATCC). The cells were incubated with Dulbecco’s Modified Eagle’s Medium (DMEM) supplemented with 10% fetal bovine serum, 1% penicillin-streptomycin, and 10 µg/mL insulin. Each of the reagents and chemicals used in the experiment was acquired from Sigma (St. Louis, MO, USA).

##### In Vitro Cell Viability Assay

The MTT assay was adopted to evaluate the prepared formula’s cytotoxic effect on skin cancer. Cells were planted in a 96-well plate at a density ranging from 1.2 to 1.8 × 10,000 cells per well. Each well received 100 µL of complete culture medium and 100 µL of the formula being tested. Following a 24 h incubation period, 100 µL of MTT was incorporated, and the mixture was incubated for an additional 4 h. Subsequently, one mL of MTT T solubilization solution was added to dissolve the formazan crystals, resulting in a purple shade [56].

##### Apoptosis Assay

To study the apoptosis process in human cancer cell line (A-431), a dual staining with fluorescein isothiocyanate (FITC)-annexin V and propidium iodide (PI) kit (Sigma-Aldrich, St. Louis, MO, USA) was conducted. The data were acquired by a Beckton Dickinson (BD) FACSCalibur™ flow cytometer (San Hose, CA, USA). The density of the cell culture was 100,000 cells/mL, and it was incubated for 24 h. Cells were then treated with the prepared formulas for 24 h as per the methods reported by M. Alyami et al. (2023) [6].

##### Cell Cycle Study

The analysis of the A-431 cell cycle after treatment with the examined formulas was assessed according to the methods reported by M. Alyami et al. (2023) [6]. Fluorescence-activated cell sorting (FACS) was used to detect the absorption of PI in A-431 cells at a density of 100,000 cells/mL for cell cycle stage determination. The samples were introduced to the cells for 24 h prior to their DNA content analysis by the BD FACSCalibur™ flow cytometer (Becton, Dickinson and Company, San Jose, CA, USA).

#### 3.2.10. In Silico Study of TRB-Loaded NEF

Molecular docking investigations were conducted according to the methodology performed by M. E. Mohamed et al. (2022) [57]. The molecular docking was performed using triangle matcher placement method and London dG scoring function. The study was carried out on the cyclin-dependent kinase 2 (CDK 2), a protein reported to be inhibited by TRB and known to regulate the cell proliferative pathway. It was executed using Molecular Operating Environment (MOE) software, version 2022.02 (Chemical Computing Group, Montreal, QC, Canada), and Discovery Studio visualizer was used for visualization (licenses number: 7486e2014469_BIO_FLEX00000004111_0001_1.lic) (Accelrys, Inc, San Diego, CA, USA). Firstly, the three-dimensional (3D) crystal structure of CDK 2 was downloaded from the protein data bank (PDB: 6GUE, https://www.rcsb.org/structure/6GUE) accessed on 30 January 2024; then, the protein structure was prepared by adding protons, eliminating unneeded water molecules, and repairing missing chains. CDK 2’s binding site was determined by analyzing the co-crystallized ligand position. A validation process was executed to verify the docking process. After constructing the two-dimensional (2D) structure of TRB using ChemDraw Professional^®^ 16.0, ligand preparation and energy reduction were performed for docking into the CDK 2 binding site. The docking scores were analyzed, and the best positions were chosen.

#### 3.2.11. Statistical Analysis

The result was analyzed statistically utilizing SPSS statistics^®^ 20 software. The analysis was conducted using Student’s *t*-test and one-way ANOVA, followed by Tukey’s post hoc test to assert the data’s significant effect (*p* < 0.05). The average data are presented as mean ± SD (*n* = 3).

## 4. Conclusions

We efficiently integrated TRB into an NE system comprising peppermint oil as the oily phase, Tween 20 as a surfactant, and ethanol as a co-surfactant in a promising skin cancer therapy. The D-optimal design was utilized to optimize the NE formulation. The optimum TRB-loaded NE formula produced a tiny, rounded droplet with an acceptable ZP value and significantly improved the cumulative TRB release compared to the plain drug. The NEF system was successfully produced, where the optimum NE formula was then integrated into a liquid foam base to prepare the TRB-loaded NEF. The developed NEF system slowed the TRB release rate in a sustained manner. The evaluation of the produced NEF formula showed that the foam system was stable. This stability was reinforced by the uniformly distributed, small bubble size, resulting in a prolonged time needed for foam collapse. Moreover, the formulated NEF-loaded TRB exhibited superior physical properties and notably enhanced the transdermal transport of TRB via rabbit abdominal skin compared to the control foam-containing TRB. Cytotoxicity study demonstrated a 10-fold significant enhancement in cytotoxicity and a 1.54-fold rise in apoptotic cell death in the A-431 cell line when treated with the optimum formula compared to plain TRB. The docking studies against CDK 2 showed that the binding scores of TRB were very promising compared to the reference drug. Based on the data obtained from in vitro and ex vivo evaluations, we suggest that TRB-loaded NEF represents a promising strategy for repurposing TRB as a topical treatment for skin cancer. However, to fully assess its therapeutic efficacy and translational relevance, future studies involving in vivo tumor suppression models are recommended.

## Figures and Tables

**Table 1:** The investigated dependent and independent variables for TRB-loaded NE preparation.

Factors	Actual Levels (Coded)	
	Low Limit (−1)	High Limit (+1)
A: oil concentration (%)	10	20
B: Smix concentration (%)	22	88
Responses	Goals	
Y_1_: PS (nm)	Minimize	
Y_2_: ZP (mV)	Maximize	
Y_3_: DE% (%)	Maximize	

Abbreviations: Smix, surfactant/co-surfactant mixture; PS, particle size; ZP, zeta potential; DE%, dissolution efficiency.

**Table 2:** Twelve TRB-loaded NE formulations based on D-optimal design asserts.

F	A: Oil Concentration (%)	B: Tween 20 Concentration in the Smix (%)	Y_1_: PS (nm)	Y_2_: ZP (mV) *	Y_3_: DE% (%)
1	20	22	173.40 ± 2.51	9.37 ± 0.97	71.51 ± 1.67
2	17	76	188.20 ± 3.41	8.16 ± 0.88	55.60 ± 0.92
3	16	51	180.10 ± 2.31	12.30 ± 1.57	79.78 ± 1.89
4	10	68	171.50 ± 1.71	11.90 ± 0.98	58.73 ± 1.67
5	20	88	157.70 ± 2.81	6.88 ± 0.57	55.16 ± 1.33
6	13	88	123.10 ± 1.91	7.26 ± 0.50	68.30 ± 1.95
7	10	22	186.60 ± 2.84	13.90 ± 0.99	86.50 ± 1.78
8	20	58	195.30 ± 3.31	6.60 ± 1.37	66.66 ± 1.34
9	10	22	180.10 ± 2.91	15.90 ± 0.77	88.50 ± 1.84
10	15	22	169.30 ± 2.11	10.50 ± 0.52	76.41 ± 1.74
11	20	22	146.90 ± 3.01	10.20 ± 0.47	75.80 ± 1.54
12	20	88	164.20 ± 2.81	7.09 ± 0.87	60.20 ± 0.80

Abbreviations: Smix, surfactant/co-surfactant mixture; PS, particle size; ZP, zeta potential; DE%, dissolution efficiency; * ZP values are expressed as absolute values.

**Table 3:** Observed and predicted values of the optimum TRB-loaded NE formula.

Factors	Optimized Level		
A: oil concentration (%)	10%		
B: Tween 20 concentration in the Smix (%)	22%		
Responses	Observed	Predicted	Prediction error (%)
Y_1_: PS (nm)	186.60 ± 2.84	182.80	−2.07
Y_2_: ZP (mV)	−13.90 ± 0.99	−14.20	2.11
Y_3_: DE% (%)	86.50 ± 1.78	83.70	−3.34

Abbreviations: PS, particle size; ZP, zeta potential; DE%, dissolution efficiency.

**Table 4:** Ex vivo permeation parameters of TRB from either TRB-loaded control foam or TRB-loaded NEF.

Parameters	TER-Loaded NEF	TER-Loaded Control Foam
Jss (μg/cm^2^·h^−1^)	47.53 ± 4.98	6.39 ± 1.83
Kp × 10^−3^ (cm/h)	19.01 ± 2.96	2.55 ± 0.87
Tlag (h)	1.91 ± 0.37	-
Er	7.43	-

Abbreviations: Jss, steady-state drug flux; Kp, permeability coefficient; Tlag, lag time; Er, the enhancement ratio.

## Data Availability

The authors confirm that the data supporting the findings of this study are available within the article and the Supplementary Materials.

## Supplementary Materials

The following supporting information can be downloaded at https://www.mdpi.com/article/10.3390/ph18070972/s1: Figure S1. In vitro cumulative release profiles of the TRB-loaded NE formulas (F1–F6); Figure S2. In vitro cumulative release profiles of the TRB-loaded NE formulas (F7–F12); Table S1. ANOVA results of the model for the three dependent variables for the TRB-loaded NE preparation; Table S2. PDI values of the TRB-loaded NE formulas.
