# Supplementary material for: Development of a Novel Drug Delivery System “Nanoemulfoam” for Topical Delivery of Terbinafine Hydrochloride as a Repurposed Therapy in Skin Cancer: Formulation, Optimization, In Vitro Characterization, Ex Vivo Transdermal Permeability, Cytotoxicity Studies, and In Silico Assessment
Source: Pharmaceuticals (Basel). 2025 Jun 27;18(7):972. doi: 10.3390/ph18070972 (PMC12300454; doi:10.3390/ph18070972)
Supplement: Supplementary file 1 [file pharmaceuticals-18-00972-s001.zip › pharmaceuticals-3649525-supplementary.pdf]

Supplementary materials

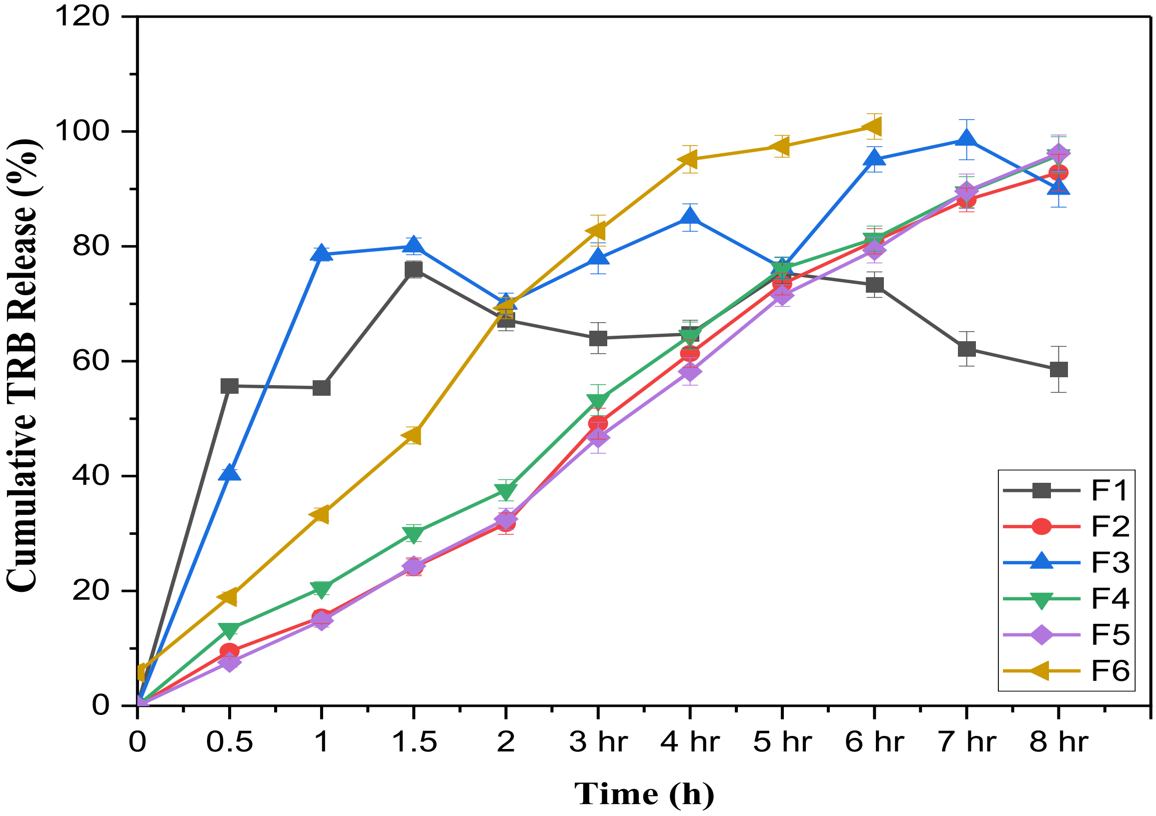

Figure S1. In-vitro cumulative release profiles of the TRB-loaded NE formulas (F1-F6).

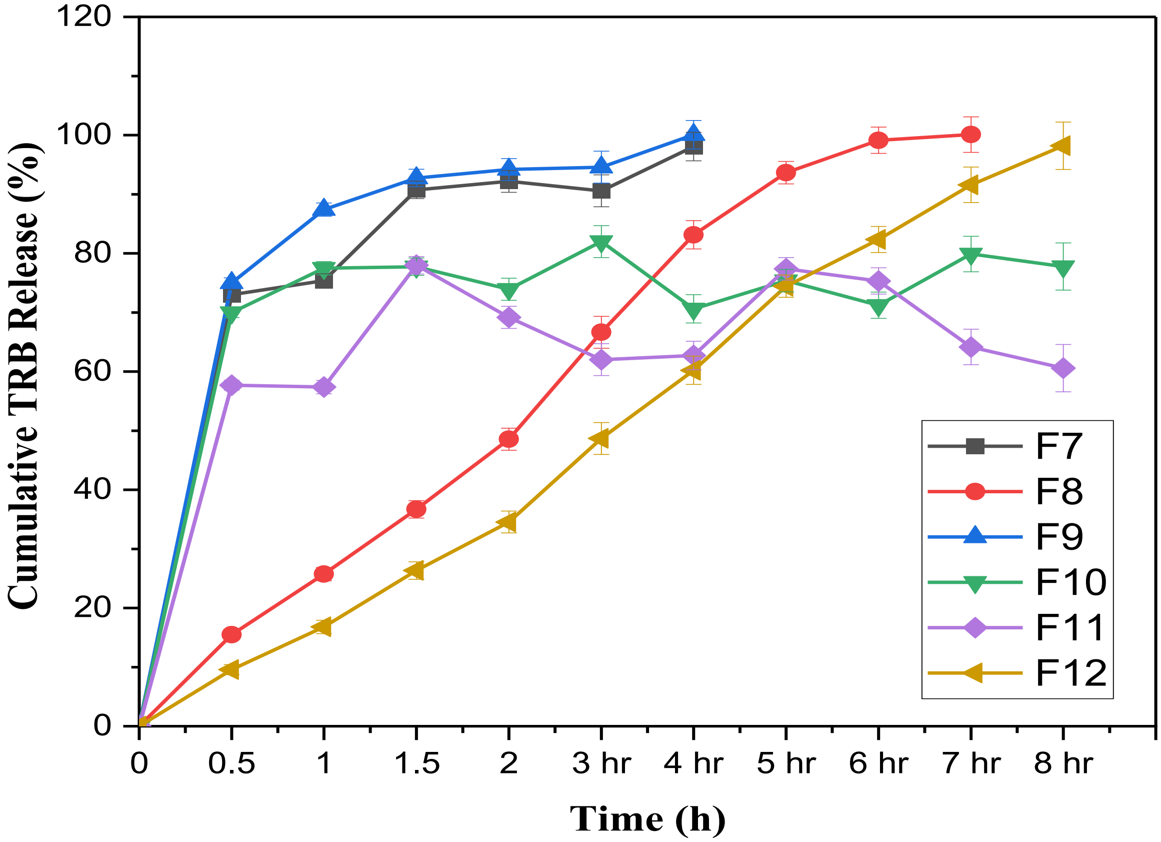

Figure S2. In-vitro cumulative release profiles of the TRB-loaded NE formulas (F7-F12).

Table S1. ANOVA results of the model for the three dependent variables for the TRB-loaded NE preparation

**Source**	Y_1: PS (nm)		Y_2: ZP (mV)*		Y_3: DE% (%)	
**Model type**	Quadratic model		Linear model		Linear model	
	**P-Value**	**F-ratio**	**P-Value**	**F-ratio**	**P-Value**	**F-ratio**
**Model**	0.0487	4.44	0.0004	20.86	0.0025	12.48
**A**	0.3440	1.06	0.0019	18.67	0.1364	2.67
**B**	0.0224	9.33	0.0043	14.31	0.0021	18.23
**AB**	0.0249	8.82	-	-	-	-
**A^2**	0.7749	0.0895	-	-	-	-
**B^2**	0.0084	14.89	-	-	-	-
Lack of Fit	0.4	1.37	0.18	3.26	0.0629	7.54
R^2 analysis						
**R^2**	0.78		0.82		0.73	

**Adjusted R^2**	0.61	0.78	0.67
**Predicted R^2**	0.25	0.70	0.52
**Adequate precision**	7.6	11.56	8.35

Abbreviations: Smix, surfactant/co-surfactant mixture; PS, particle size; ZP, zeta potential; DE%, dissolution efficiency.

Table S2. PDI values of the TRB-loaded NE formulas.

F	A: oil concentration (%)	B: Tween 20 concentration in the S_{mix} (%)	PDI
**1**	20	22	0.44 ± 0.075
**2**	17	76	0.45 ± 0.065
**3**	16	51	0.53 ± 0.075
**4**	10	68	0.51 ± 0.085
**5**	20	88	0.56 ± 0.095
**6**	13	88	0.72 ± 0.055
**7**	10	22	0.61 ± 0.023
**8**	20	58	0.70 ± 0.044
**9**	10	22	0.60 ± 0.045
**10**	15	22	0.61 ± 0.023
**11**	20	22	0.41 ± 0.025
**12**	20	88	0.53 ± 0.048
